# Lyme Borreliosis as a Trigger for Autoimmune Disease

**DOI:** 10.7759/cureus.18648

**Published:** 2021-10-10

**Authors:** Yelyzaveta Yehudina, Svitlana Trypilka

**Affiliations:** 1 Rheumatology, Institute of rheumatology, Kyiv, UKR; 2 Rheumatologist Policlinic, Communal Non-Commercial Enterprise of Kharkov Regional Council "Regional Clinical Hospital", Kharkiv, UKR

**Keywords:** systemic lupus erythematosus, diagnosis, trigger, autoimmune disease, lyme disease

## Abstract

Lyme disease (LD), also known as Lyme borreliosis, is a zoonotic disease caused by the Gram-negative bacteria Borrelia burgdorferi sensu lato belonging to the Spirochaetaceae family. Differentiating LD from other systemic disorders that present with musculoskeletal symptoms is challenging, and the presence of antibodies to borrelia in the general population may contribute to misdiagnosis. Moreover, long-term exposure of the host’s immune system to spirochetes can contribute to the development of chronic autoimmune disease de novo. We report a 35-year-old woman with a combination of LD and systemic lupus erythematosus (SLE), and in this case, LD was the most likely trigger for SLE. We also performed a literature review and summarized the previously reported cases with a combination of LD and autoimmune disease.

## Introduction

Autoimmune diseases are typically characterized by multisystem involvement and a wide range of symptoms, which poses a challenge in the differential diagnosis and management of these patients. Systemic lupus erythematosus (SLE) is one of the most severe connective tissue systemic diseases, with some atypical variants (with respect to onset and course) that are resistant to aggressive treatment.

Lyme disease (LD), also known as Lyme borreliosis, is a zoonotic disease caused by the Gram-negative bacteria Borrelia burgdorferi sensu lato (B. burgdorferi s.l.) belonging to the Spirochaetaceae family. Differential diagnosis of LD is often difficult since the symptoms may mimic a wide range of systemic diseases with musculoskeletal symptoms. In addition, the high prevalence of antibodies against borrelia in the general population may contribute to misdiagnosis.

LD may present with a wide range of clinical manifestations including symptoms of dermatological, articular, nervous, cardiovascular, cardiac, and ocular involvement. It should be noted that these are typical symptoms of many autoimmune diseases, particularly SLE. The intersection of these two pathologies in clinical practice is of considerable interest in view of the need to differentiate the main pathology and determine the management strategy.

The pathogenesis of LD in the early stages is largely associated with the presence of viable bacteria at the site of inflammation, while in the later stages of the disease, the clinical manifestations and the involvement of organs and systems are largely due to the autoimmune mechanisms. Long-term exposure of the host’s immune system to spirochetes can contribute to the de novo development of chronic autoimmune disease [1]. For rheumatologists, the intersection of these two diseases may be of clinical interest, since the symptoms of LD can mimic many other illnesses and have been linked to several autoimmune diseases.

In this study, we report a patient who developed SLE a few months after being treated for LD. In addition, we conducted a literature search for previous studies reporting the development of autoimmune disease after LD. The findings of the review are summarized along with a discussion of the underlying mechanism of the association.

## Case presentation

A 35-year-old woman was referred to the rheumatologist in October 2020 with complaints of pain in the hand joints, episodes of low-grade fever, skin rashes over the hand and trunk, general weakness, and fatigue. In August 2020, she had sustained an insect bite after which she developed changes in skin color (purple-cyanotic areas) and skin thickening on the right lateral surface of the trunk and right buttock. Subsequently, she noticed a gradual onset of general weakness, pain in the hand joints, episodes of increased temperature (up to 37.2°C-37.5°C), and a change in the color of the skin over the hand (Gottron's sign - violaceous erythema over the back of the fingers). The patient consulted a dermatologist, who prescribed topical treatment (ointment containing steroids) for dermatitis for one month but with no response. Owing to the worsening of her condition (increase in body temperature and articular syndrome), she consulted the rheumatologist.

**Figure 1:**
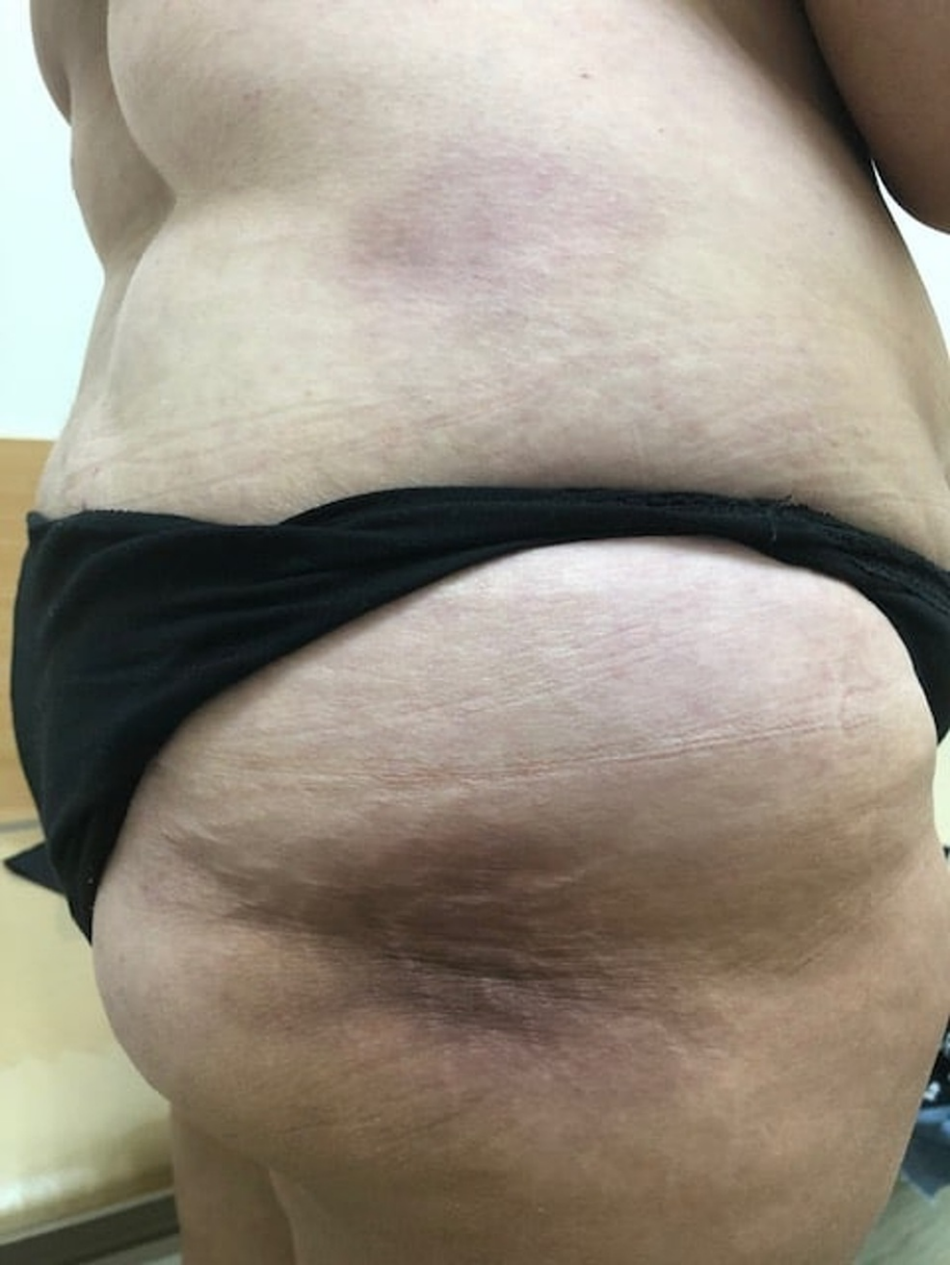
Patient’s skin changes: a rounded purple-cyanotic painless dense formation is seen on the right lateral surface of the trunk and buttock.

**Figure 2:**
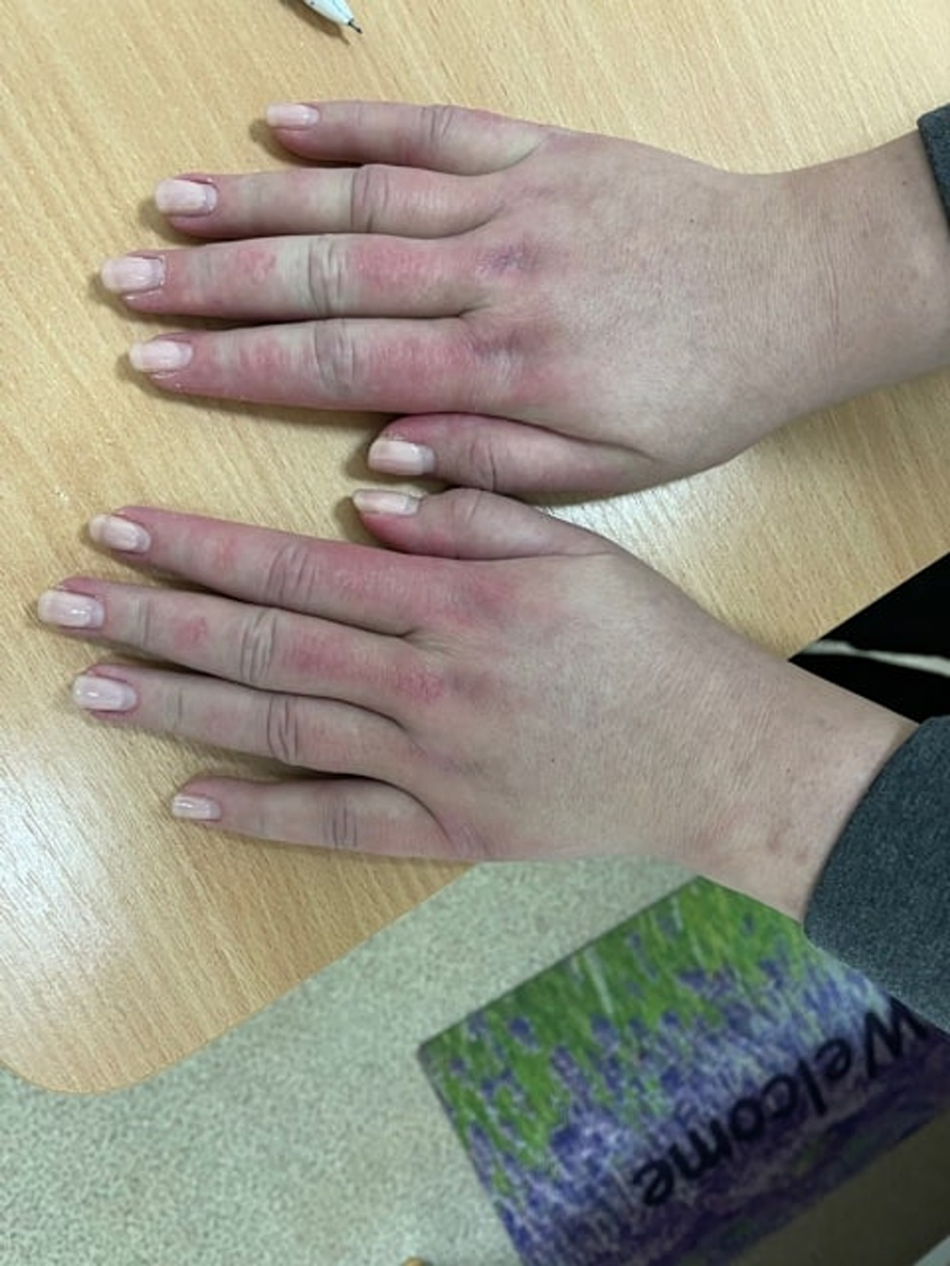
The skin of the hand showing Gottron's sign.

Skin examination revealed a rounded purple-cyanotic painless dense formation, which was difficult to plicate, on the right lateral surface of the trunk and buttock, and poikiloderma of the hand skin (Figures 1, 2).

Series of clinical blood tests conducted between October 2020 and February 2021 showed the following results: leukopenia (WBC count range: 3.07-2.67 × 10^9^/L); increased erythrocyte sedimentation rate (range: 33-39 mm/h); C-reactive protein (CRP) 48 mg/L, glomerular filtration rate 92 mL/min/1.73m^2^, proteinuria (24-hour urinary protein: 0.5 g/L).

Considering the history of a tick bite, skin changes, and articular syndrome, the patient underwent serological testing for Lyme borreliosis. Enzyme immunoassay and Western blot analysis were positive for specific anti-Borrelia burgdorferi IgG and IgM (IgG and IgM ≥ 1.1; Ig M OspC, p 41, p39, p23; Ig G VlsE, р41- positive).

The patient was diagnosed with LD and was prescribed antibiotic therapy with doxycycline 100 mg, twice per day orally for 28 days. Following therapy, her general condition improved with resolution of joint pain, weakness, normalization of body temperature, and decrease in rashes.

However, at the end of January 2021, i.e., two months after the antibiotic therapy, the patient presented again with episodes of increased body temperature (peak: 38.3°C), butterfly-like bilateral erythema on the cheek, diffuse alopecia, pain, and morning stiffness in the metacarpophalangeal, interphalangeal and wrist joints lasting more than two hours, weight loss of 4 kg in a month, pronounced chilliness of the fingers. Taking into account the change in the clinical manifestations, further investigations were conducted to exclude systemic connective tissue diseases and lymphoma.

**Figure 3:**
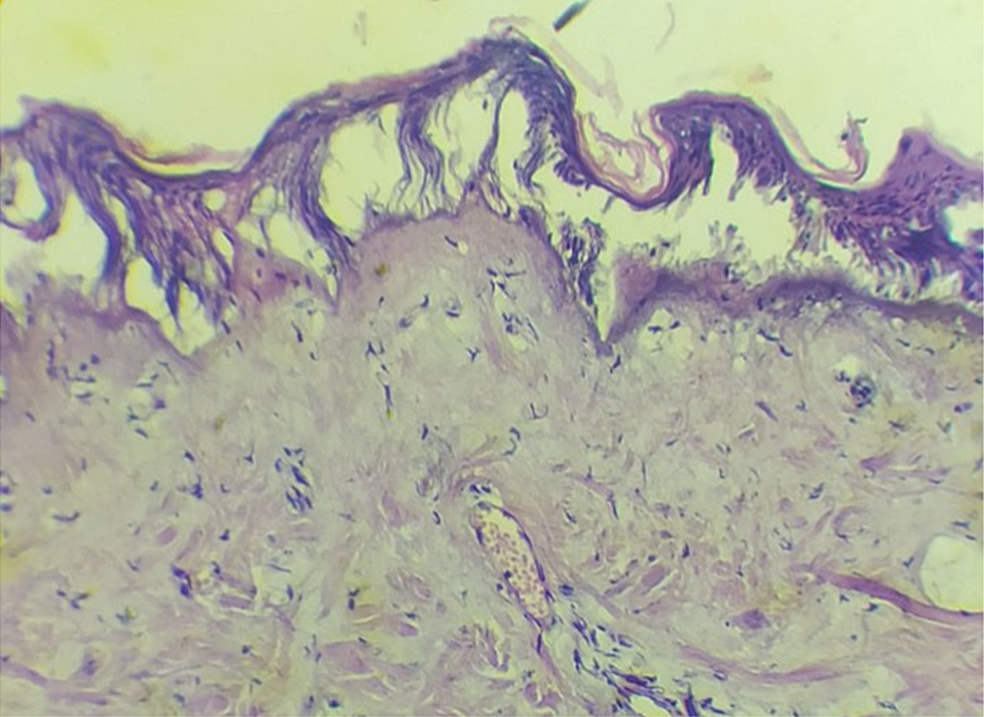
Biopsy of the skin showing light microscopy appearance of vasodilation in the papillary layer, perivascular lymphocytic infiltrates around the vessels in the subepithelial dermis (hematoxylin-eosin staining, 100×, 200×).

**Figure 4:**
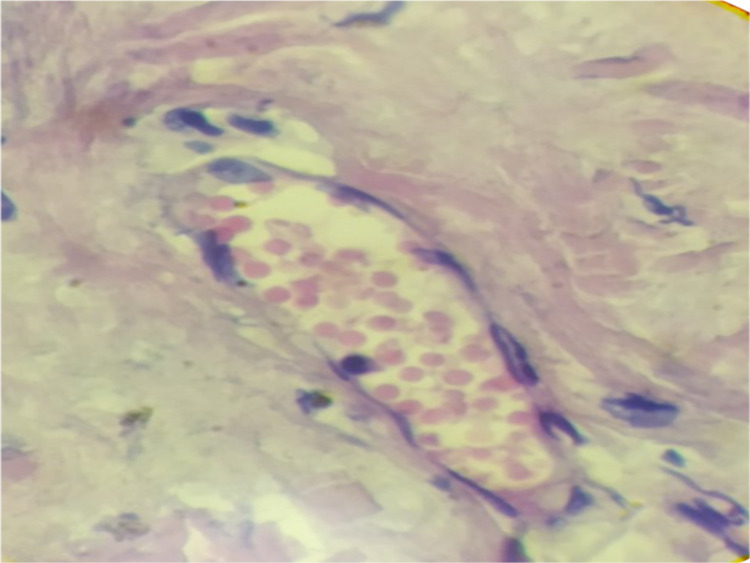
Biopsy of the skin showing light microscopy appearance of erythrocytes and mononuclear cells in the vascular lumens (hematoxylin-eosin staining, 100×, 200×).

The patient underwent a biopsy of a skin lesion with a depth and width of 2 cm (Figures 3, 4). Direct immunofluorescence study revealed large-globular deposits of immunoglobulins in the basement membrane. In the subepithelial dermis, there was vasodilation of the papillary layer, with no pronounced perivascular lymphocytic infiltrates (not an acute phase); erythrocytes and mononuclear cells were observed in the vascular lumen. The biopsy impression was hyperkeratosis, thinning, atrophy of the epidermis with vacuolar degeneration of the basal layer and detachment of the squamous epithelial layer.

Tests for some immunological markers were performed: antinuclear antibodies (ANA), 1: 320 (positive), homogeneous pattern; anti-nRNP, 26 U/mL (positive); compliment C3 and C4, normal; antibodies to double-stranded DNA and anti-Smith - negative; lupus anticoagulant was detected, screening test 65.8 sec (31.0-44.0), confirmation test 39.8 (30.0-38,0); LA-AUTO 1.6533 (1.5-2.0, moderate risk).

**Figure 5:**
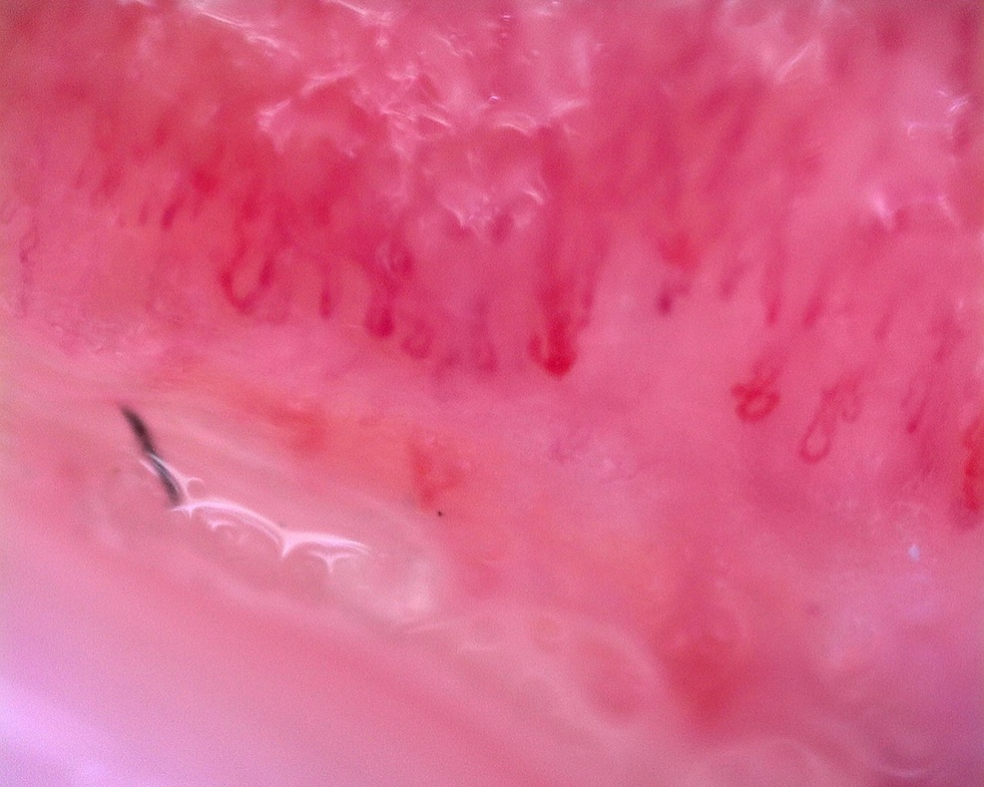
Nailfold capillaroscopy showing active scleroderma pattern, avascular zones, decreased density of capillary loops, and giant capillary (200×).

Nailfold capillaroscopy was performed to differentiate the genesis of Raynaud’s phenomenon (Figure 5). When analyzing the capillary blood flow, attention was paid to significant disorganization of the capillary network, multidirectional capillary loops, and microhemorrhages. The shape of the capillary loops was atypical: crossed, twisted, branched. Expanded giant capillaries, bush-like were observed, which indicated neoangiogenesis. Capillaries with elements of arteriolospasm, expansion of the venular segment, and stasis in the transitional areas were found. Venous plexus and venulo-venular anastomoses were visualized. The specific gravity was reduced, indicative of avascular zones. The capillaroscopic picture was consistent with a scleroderma-like pattern and spastic type of microcirculation.

The clinical data and the presence of an entry criterion (ANA titer 1: 320) allowed us to diagnose SLE (20 points: fever, subacute cutaneous lupus, polyarthritis, proteinuria, alopecia, high lupus anticoagulant) according to the current diagnostic criteria EULAR/ACR 2019 [2].

Therefore, prednisolone (PZ) 40 mg per day, hydroxychloroquine (HCQ) 400 mg per day, calcium, and vitamin D were prescribed. At a three-month follow-up, there was a significant improvement in the general condition of the patient, i.e., general well-being, a temperature within the normal range, significant improvement in articular syndrome, and decreased intensity of facial rashes. The patient was advised to continue the prescribed treatment with regular follow-up examination.

We searched the literature for a similar clinical problem and identified only seven case reports and one cohort of patients with Lyme borreliosis-related autoimmune disease development.

## Discussion

LD is a multisystem disease that has a wide variety of manifestations [1]. It is a complex immune-mediated disease, infectious in origin and inflammatory in nature. Long-term exposure of the host’s immune system to spirochetes can cause chronic autoimmune disease. Interaction of the bacterium with the host results in pro-inflammatory, immunomodulatory, and immunosuppressive effects induced by the pathogen. One possible explanation for antibiotic-resistant LD or subsequent autoimmune reactions and diseases is the generation of autoimmunity directly or indirectly mediated by the pathogen and based on molecular mimicry [3].

There is strong evidence of the presence of an immune-mediated process in patients with antibiotic-resistant LD. This outcome is associated with certain human leukocyte antigen (HLA)-DR alleles, excessive joint inflammation, immune dysregulation of the CD41 Teff:Treg ratio of cells, and infection-induced autoimmunity [4].

Genetic linkage studies in adults with Lyme arthritis have shown a link with major histocompatibility complex (MHC) class II DR2 and DR4 molecules [5]. MHC class II molecules play a critical role in the activation of the immune system. Polymorphism in the genes that make up the MHC class II structure affects the immune system in at least two ways. First, polymorphic amino acid residues on individual class II proteins determine whether a particular peptide will bind, and, therefore, appear as a particular class II molecule displayed on an antigen-presenting cell. Second, MHC class II molecules regulate the ontogenetic selection of the T-cell receptor specificity in the thymus, thereby affecting the repertoire of CD8+ and CD4 + T cells that recognize foreign peptides in the context of MHC class II molecules [6]. These studies suggest that Borrelia antigens are loaded onto MHC class II molecules and subsequently presented to CD4 + T lymphocytes [7].

Studies have reported some similarities between the musculoskeletal manifestations of LD and rheumatoid arthritis. The latter has an autoimmune component and is associated with the HLA system, which encodes class II proteins of the MHC, in particular the HLA-DR isotype [8]. Studies have investigated the relationship of certain serotypes of HLA-DR with the predisposition of patients with LD to develop chronic arthritis. In a study of 130 patients with various manifestations of LD [9], HLA-DR4 was found to be more common in patients with chronic arthritis and HLA-DR4 was the only serotype that showed a significant association with treatment-resistant Lyme arthritis. Overall, 89% of patients with chronic arthritis had either HLA-DR4 or -DR2, while some of the patients had both. This indicated that HLA-DR4 and -DR2 are potential markers of susceptibility to rheumatoid arthritis [9].

Infection can transform the preclinical phase of an autoimmune disease into an active one. In the preclinical stages, autoantibodies may be present for years prior to the development of overt systemic disease. Many infectious agents may serve as triggers for the initiation of clinical expression of autoimmune diseases [10]. In addition to antigen-specific activation of lymphocytes, microbes can provide a secondary signal required to induce a pathogenic autoimmune response, which is referred to as the adjuvant effect of infection [11].

A few case reports have described the relationship between LD and autoimmune disease development (Table 1). Early publications have described individual cases [12], while in recent years, the frequency of such cases has increased with several cohorts of patients having been reported. Few cases of dermatomyositis [13], Guillain-Barre syndrome, Sjögren’s syndrome, Still’s disease and SLE have been described.

**Table 1:** Important reports in the literature regarding autoimmune disease development after Lyme disease. ANA, antinuclear antibodies; anti-CCP, cyclic citrullinated peptide antibody; RA, rheumatoid arthritis; PsA, psoriatic arthritis; pSpA, peripheral spondyloarthritis; SLE, systemic lupus erythematosus.

Patient diagnosis	Patient age/Sex	Evidence for Lyme disease	Presentation of autoimmune disease	References
Dermatomyositis	52/male	Erythema migrans, positive immunosorbent assay, and Western blot	Periorbital edema, dysphagia, proximal muscle weakness, markedly elevated level of creatine phosphokinase	Horowitz, et al. [12]
Dermatomyositis	50/female	Tick bite, erythema migrans lesion	Fever, myalgia, paresthesia, weight loss, and motor weakness telangiectasia and periungual discoloration, markedly elevated level of creatine phosphokinase	Birlutiu, et al. [13]
Guillain-Barre Syndrome	73/female	Positive Lyme ELISA and confirmatory Western Blot analysis	Progressive numbness and weakness in lower extremities bilaterally and numbness in chest and abdomen, lips, speech impairment	Awan, et al. [14]
Guillain-Barre Syndrome	31/male	Positive Lyme disease immunoblot positive	Progressive numbness and weakness in bilateral hands and feet, areflexia, decreased sensation, and numbness in the tongue	Patel, et al. [15]
Sjögren’s syndrome	43/female	Tick bite, positive enzyme immunoassay, and Western blot analysis	Keratoconjunctivitis sicca; xerostomia; positive tests for antinuclear antibodies, anti-Ro (SSA), anti-La (SSB), anti-SS-A, and anti-SS-B IgG antibodies;	Smiyan, et al. [16]
Still’s disease with hemorrhagic pericarditis and tamponade	61/male	Erythema migrans	Acute on chronic fibrinous hemorrhagic pericarditis, adult-onset Still’s disease	Ocon, et al. [17]
RA (n = 15)	55 (24–70) 9/6 male/female	Erythema migrans	Polyarticular (5 + joints), RF, and/or anti-CCP positive	Arvikar, et al. [18]
PsA (n = 13)/ pSpA (n = 2)	48 (18–73) 9/6 male/female	Erythema migrans	Axial disease, enthesitis, dactylititis	Arvikar, et al. [18]
SLE	39/female	Tick bite, local erythema and later migrating skin change, positive Western blot analysis	Lymphopenia, increasing titers of anti-ds-DNA antibodies, and renal involvement as erythrocyturia and proteinuria	Federlin and Becker [19]
SLE	32/female	Tick bite, erythema migrans lesion, positive enzyme immunoassay, and Western blot analysis	Fever, subacute cutaneous lupus, polyarthritis, proteinuria, alopecia, high lupus anticoagulant, positive ANA	Current case

The largest cohort of patients with the development of post-Lyme autoimmune disease has been described by Dr. Arvikar and colleagues [18]. They identified 30 patients (median age: 55 years) who developed the new-onset systemic autoimmune joint disease at a median interval of four months after the diagnosis of LD; in their cohort, 15 patients developed rheumatoid arthritis, 13 developed psoriatic arthritis, and two developed peripheral spondyloarthritis.

Our patient showed a gap of several months between infection and development of systemic autoimmune disease, which may reflect the time required for affinity maturation or epitope propagation of autoimmune responses. Although spirochetal infection itself may be a trigger, disruptions in the microbiome resulting from antibiotic treatment of B. burgdorferi infection may also be an underlying mechanism [20]. There is evidence in a person with a genetic predisposition to autoimmune diseases (such as SLE or rheumatoid arthritis), infection with B. burgdorferi can trigger their development [21]. Further research on HLA-DR and dendritic cell response to B. burgdorferi may help develop new treatments for LD and contribute to a better understanding of the mechanism of causation of autoimmune diseases.

## Conclusions

The combination of LD and autoimmune disease is rarely encountered in clinical practice and is difficult to treat. The condition is of greatest interest for rheumatologists, infectious disease specialists, and dermatologists. Owing to the rarity of this pathology, patients may not receive the proper attention of a multidisciplinary team for a long time. Moreover, the overlapping symptoms of these diseases, the difficulty of distinguishing between these two pathologies, and the possibility of relapse of both conditions are a challenge for treatment decision-making. The advisability of antibiotic therapy and disease-modifying antirheumatic drugs in the presence of alleviation of clinical symptoms at the onset of the disease may delay the timely initiation of adequate treatment.
